# Anisotropic Alteration of Scleral Birefringence to Uniaxial Mechanical Strain

**DOI:** 10.1371/journal.pone.0058716

**Published:** 2013-03-11

**Authors:** Satoko Nagase, Masahiro Yamanari, Ryosuke Tanaka, Takeshi Yasui, Masahiro Miura, Takuya Iwasaki, Hiroshi Goto, Yoshiaki Yasuno

**Affiliations:** 1 Department of Ophthalmology, Tokyo Medical University, Ibaraki Medical Center, Ami, Ibaraki, Japan; 2 Computational Optics and Ophthalmology Group, Tsukuba, Ibaraki, Japan; 3 Computational Optics Group, University of Tsukuba, Tsukuba, Ibaraki, Japan; 4 Tomey Corporation, Nagoya, Aichi, Japan; 5 Graduate School of Engineering Science, Osaka University, Toyonaka, Osaka, Japan; 6 Institute of Technology and Science, University of Tokushima, Tokushima, Tokushima, Japan; 7 Department of Ophthalmology, Tokyo Medical University, Shinjuku, Tokyo, Japan; Dalhousie University, Canada

## Abstract

**Purpose:**

To investigate the relationship between scleral mechanical properties, its birefringence, and the anisotropy of birefringence alteration in respect of the direction of the strain by using PS-OCT.

**Methods:**

The scleral birefringence of thirty-nine porcine eyes was measured with a prototype PS-OCT. A rectangle strip of sclera with a width of 4 mm was dissected at the temporal region 5 mm apart from the optic nerve head. The strain and force were measured with a uniaxial tension tester as the sample was stretched with a speed of 1.8 mm/min after preconditioning. The birefringence of the sample was measured by PS-OCT at the center of the sample before applying, denoted as inherent birefringence, and after applying stretching of 6.5% strain. The birefringence alteration was obtained by these two measurements and correlations between birefringence and elastic parameters, tangent modulus, and structural stiffness were examined. Twenty and 19 porcine eyes were stretched in meridional or equatorial directions, respectively.

**Results:**

A moderate positive correlation was found between the inherent birefringence and the structural stiffness. A moderate positive correlation was also found between the inherent birefringence and the tangent modulus. The birefringence increased by strains. Marginal significance was found in the birefringence alteration between meridional and equatorial strains, where the mean birefringence elevation by meridional strain was higher than that by equatorial strain.

**Conclusions:**

The birefringence was found to be altered by applying strain and also be related with inherent birefringence. This implies the birefringence of the sclera of the *in vivo* eye also could be affected by its mechanical property.

## Introduction

Glaucoma is the second leading cause of blindness in the world[1]. It is a progressive optic neuropathy due to loss of retinal ganglion cells and the retinal nerve fiber layer that comprises axons of these cells, and is characterized by optic nerve damage and typical visual field loss [2]. The diagnosis of glaucoma is based on characteristic optic nerve cupping which corresponds with the visual field defect. However, it has been found that there is a loss of approximately 40% of retinal ganglion cells before visual field damage is detected [3]. Because glaucoma damage is irreversible, early detection and appropriate management of progressive changes are the best ways to prevent loss of visual function. In the diagnosis of glaucoma, structural loss can precede detectable functional loss by up to 5 years [4], and the glaucomatous retinal nerve fiber layer defect can be detected morphologically by red-free fundus photographs earlier than white-on-white visual field defects [5].

Optical coherence tomography (OCT) is an emerging technology for high resolution, noncontact imaging in transparent and translucent structures[6], which has proven useful for measuring circumpapillary nerve fiber layer thickness for glaucoma detection. This modality can detect circumpapillary nerve fiber thickness loss with good sensitivity and specificity. However, since the glaucoma damage of nerve fiber is irreversible, it is important to investigate an earlier signature of glaucoma than the nerve fiber loss.

It has been known that the posterior sclera plays a significant role in the development of glaucoma, and it has been suggested that damage due to intraocular pressure (IOP) can be affected by the mechanical properties of this structure. Relatively low IOP can itself lead to increased posterior deformation in the presence of altered scleral mechanical properties [7].

Human scleral tissue is comprised of approximately 50% collagen by weight, consisting predominantly of type 1 collagen [8]. Type 1 collagen involves formation of subfibrillar units that assemble to form fibrils and bundles of fibrils, and these fibril units may be important in detecting the mechanical properties of collagen fibers [9]. In addition, these fibrils are known to be aligned with some characteristic directionality [10]–[14].

We have previously shown a correlation between scleral birefringence and elasticity using *ex vivo* porcine sclera [15]. This correlation could be explained by the fact that both factors are strongly related to highly organized collagen fibers such that the birefringence of collagen fiber assembly is related to the scleral microscopic properties. The highly organized collagen makes the anisotropy of mechanical properties and also birefringence alteration simultaneously.

In the previous study [15], a custom-made, polarization sensitive optical coherence tomography (PS-OCT) [16] was utilized to investigate scleral birefringence. PS-OCT is one of the functional extensions of OCT which is capable of imaging the birefringence of biological fibrous tissue [17]. Hence, PS-OCT is expected to be suitable for indirect investigation of scleral microstructure.

In the previous study, correlation between scleral birefringence and mechanical properties was investigated in the relaxed state. However, lack of knowledge about scleral birefringence under mechanical force/tension limits our understanding of the mechanism behind this correlation. In this paper, we further investigated the relation between scleral mechanical property and birefringence. The scleral birefringence of the sclera is measured both at the relaxed state and under strain. In addition, the anisotropy of the birefringence was investigated as functions of the direction of strain. Based upon these results, PS-OCT could potentially be developed as an early diagnostic tool for scleral abnormality and possible glaucoma.

## Methods

### Specimen preparation

**Figure 1:**
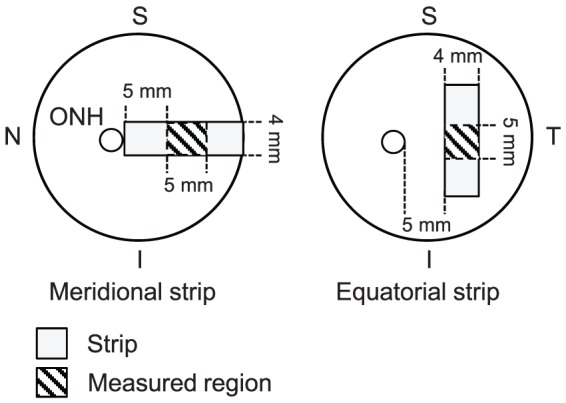
Porcine sclera dissected by scalpel blades. A strip of sclera was dissected at the temporal region 5 mm apart from ONH, resulting in a rectangular shape with a width of 4 mm and a length of approximately 15 mm.

Thirty-nine *in vitro* porcine eyes were obtained from a local abattoir (Daimon Co., Ltd., Japan) and dissected and measured within 24 hours of sacrifice. A rectangle strip of sclera with a width of 4 mm and a length of about 15 mm was dissected at the temporal region 5 mm apart from the optic nerve head (ONH) as shown in Fig. 1. The nasal region and the region near the limbus were excluded because the scleral thickness has high spatial variation at these position. During the dissection, the sample was cooled with an iced coolant.

**Figure 2:**
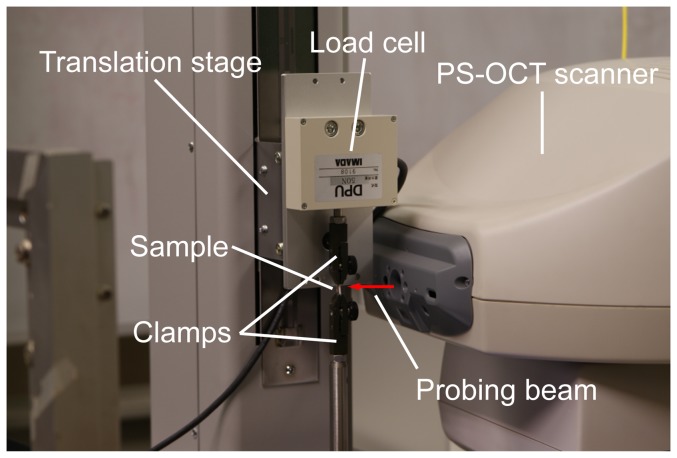
The experimental system for birefringence and elasticity measurements.

To apply strain to each scleral sample, the sample was held by a pair of clamps of a uniaxial, motorized, stretching tension tester (MX2-500N, Imada Co., Ltd., Toyohashi, Aichi, Japan) as shown in Fig. 2. The initial distance of the pair of clamps was 5 mm. The sample was stretched along the long axis of the strip, which was oriented to one of the meridional or equatorial directions. Here, the meridional direction denotes the direction parallel to the meridional line of the eye globe, and the equatorial direction denotes the direction parallel to the equator of the eye globe.

Before performing the measurements, the sample was prepared by a standard preconditioning protocol, which is later described. During the experiment, saline was applied to the tissue to keep it moist. Twenty and 19 porcine eyes were dissected for meridional stretch and for equatorial stretch, respectively.

### Measurement of cross-sectional area of sclera

**Figure 3:**
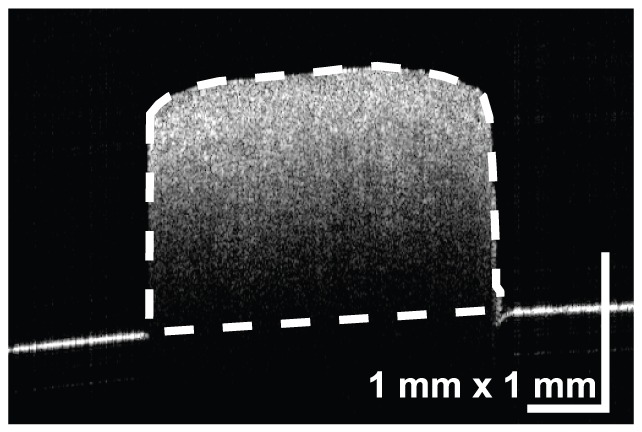
An OCT image with a rubber plate used for the measurement of the cross-sectional area. The area between the sample surface and the surface of the rubber plate behind the sclera, which is defined by line interpolation from the exposed surface of the rubber plate, was defined as the cross-sectional area.

Initially, a cross-sectional area of the sample required for the calculation of elasticity was measured by OCT. The porcine sclera is thick and the back surface of the sample could not be imaged by OCT in all samples. In addition, the refractive index of the scleral sample was not known, and it was therefore not possible to directly measure the cross-sectional area using standard OCT measurement techniques. To overcome this problem, a flat rubber plate was attached behind the sample during the OCT measurement. The surface topography was manually obtained from the OCT measurement, and the area between the sample surface and the surface of the rubber plate behind the sclera was defined as the cross-sectional area as shown in Fig. 3. The rubber surface was defined by line interpolation from the exposed surface of the rubber plate. The cross-sectional area was then measured at the center of the two clamps.

### Measurement of stress-strain response of the sclera

We measured the strain-stress response of the sample, and calculated elastic parameters, tangent modulus, and structural stiffness.

**Figure 4:**
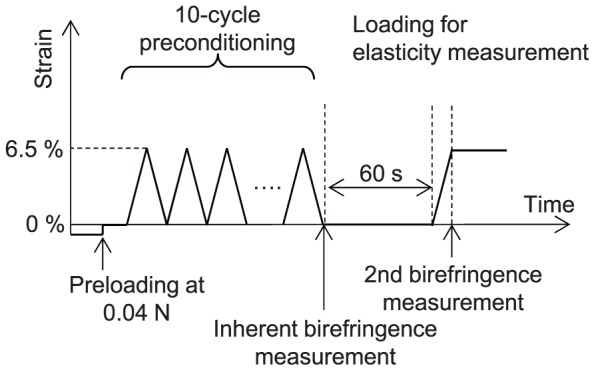
The time chart of strain applied during the measurement. Prestress of 0.04 N was applied to remove slack of the sample. Ten-cycle preconditioning was applied by loading and unloading from 0–6.5 % strain with a speed of 1.8 mm/min. After 10 s of preconditioning, the sample was extended from 0–6.5 % strain with the same speed.

The operational diagram of the motorized translation stage is shown in Fig. 4. A prestress of 0.04 N was applied to remove any slack of the sample. After removing a rubber plate, preconditioning was applied with ten cycles of loading and unloading from 0–6.5% strain with a speed of 1.8 mm/min. After 10 cycles of preconditioning, the sample was extended from 0–6.5 % strain with the same speed. The purpose of the preconditioning was to remove the variation of hysteresis in stress-strain curve among stretching cycles. The preconditioning protocol utilized in this study has been preliminarily validated for this purpose by an experiment. For sample extension, the maximum strain of 6.5% was adopted by the following reason. In previous studies of scleral biomechanics, wide range of strains from 1% to 20% was utilized. In our study, in order to mimic physiological condition, we selected one of the smallest strains which was achievable by the accuracy of our translation stage among the strains of previous studies[18]. The strain and force were measured with the uniaxial tester as the sample was stretched with a speed of 1.8 mm/min. Twenty and 19 porcine eyes were stretched in meridional or equatorial directions, respectively.

#### Calculation of elastic parameters

**Figure 5:**
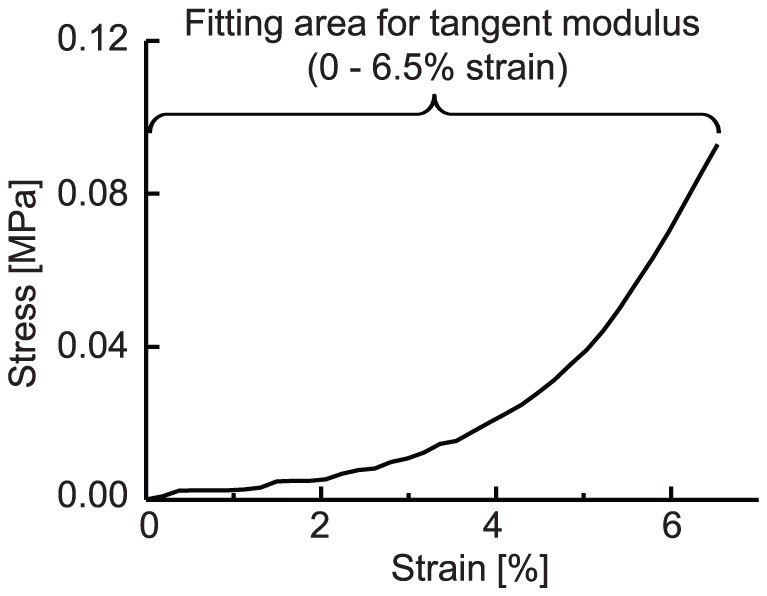
An example of stress-strain curve. The region from 0% to 6.5% strain was fitted by an analytic curve to obtain a tangent modulus.

The stress-strain curve was obtained by the uniaxial tester, and was fitted with an exponential model [19], 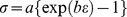, where 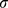 is tensile stress, which is the force applied to the sample divided by the initial cross-sectional area of the sample, and 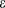 is the strain, namely, 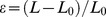 where 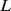 and 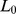 are the lengths of the sample at a certain time and the initial length of the sample, respectively. Fig. 5 shows a representative stress-strain curve of the sclera. Tangent modulus at 0 % strain 
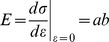
, was used for the analysis. To consider a net stiffness of sclera, structural stiffness was defined as a product of tangent modulus and thickness [20]. Power of exponential stress-strain function, which was equivalent to the slope between tangent modulus and stress, *b*, was also extracted from the fitting [19].

### Birefringence measurement

Before and after the measurement of strain-stress response of the sclera, OCT data of the sclera were acquired with a custom-made PS-OCT [16] with a probe beam light with center wavelength of 1.31 µm. The axial and lateral resolutions of the PS-OCT in the tissue were 9.2 µm and 20.5 µm, respectively. The outer surface of the sclera was positioned to face the scanner of the PS-OCT. A-scans (512×1280) covering 6×3 mm^2^ on the sample were acquired. Since collagen bundles were densely packed in sclera and the diameters of collagen bundles and fibrils were much smaller than the axial resolution of OCT, no structure was directly resolved in the sclera.

The PS-OCT provided a three-dimensional Jones matrix tomography of the sample, in which each pixel of the tomography represents the Jones matrix of the sample. To calculate the sample birefringence from the Jones matrix tomography, we developed a custom signal processing algorithm as follows. The Jones matrices were moving averaged with a kernel size of 3×5 pixels (axial × lateral) by a complex Jones matrix averaging algorithm [16]. Local roundtrip Jones matrices were calculated with a pixel separation of 8 pixels (49 µm) [21], Raw birefringence values of the sample were obtained by Lu-Chipman decomposition of the local roundtrip Jones matrices [22]. The systematic error of the birefringence was corrected by a Monte Carlo-based mean estimator.[23] The birefringence values obtained from pixels with an effective signal-to-noise ratio[21] of greater than 10 dB were utilized for subsequent analyses. Technically, the birefringence is defined as the difference of two refractive indexes corresponding to two orthogonal polarization states, and hence is unitless. However, in this study, the birefringence was expressed as the phase-delay per unit depth from the difference of the refractive indexes (i.e., degree-per-micrometer to be consistent with the convention of PS-OCT). Although the unitless representation of the birefringence and the phase-delay per unit depth have different scales, they are essentially the same and have a perfectly linear relationship, i.e., 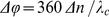 where 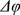 is the birefringence represented in degree-per-micrometers, 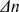 is the unitless birefringence, and 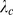 is the center wavelength of the PS-OCT probe beam in micrometers. These two representations of the birefringence therefore made no difference for subsequent statistical analyses.

As predicted from the anatomy of the sclera [11], the birefringence was not found to be uniform throughout the sample. In addition, because of our calculation methodology, the measured birefringence would result in an artifact at the boundary of tissue domains which have different optic axes. To resolve this issue, we defined a new parameter denoting the degree of optic axis uniformity (DOAU) as follows. By using Stokes parameters of eigenvectors of the Jones matrices, the DOAU is defined as 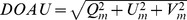, where 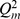, 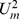, and 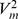 are Stokes parameters of axis orientations averaged in a kernel size of 5×11 pixels (axial × lateral, 31×129 µm^2^) centered at *m*-th pixel of the B-scan image. DOAU of zero indicated a completely random orientation, and unity indicated a completely uniform orientation in the local regions of the kernel size. In contrast to the degree of polarization uniformity [24], which is a similar quantity to DOAU, DOAU did not represent a state of polarized light, but could indicate regions with high uniformity of optic axes in the sample which have reliable local birefringence. Only the birefringence values obtained from pixels with DOAU greater than 0.9 were used for the analyses, and the mean birefringence was calculated for each volume of the sample.

The first birefringence measurement was performed right after the preconditioning at 0% strain, and this birefringence value was denoted as inherent birefringence because which would be similar to that under physiological condition. The second birefringence measurement was performed after stretching of the sample at 6.5% strain by the uniaxial tester, and alteration of the birefringence was obtained from these two measurements.

### Statistical analysis of birefringence and elastic parameters

Two-sided tests using Pearson’s product-moment correlation coefficient were applied to the birefringence and elastic parameters. A paired t-test evaluated the birefringence alteration by stretching, and analysis of covariance (ANCOVA) was used to assess the inherent birefringence and birefringence alteration between each strain direction. For the statistical calculation, statistical computing language, R ver. 2.12.2 was used.

## Results

**Figure 6:**
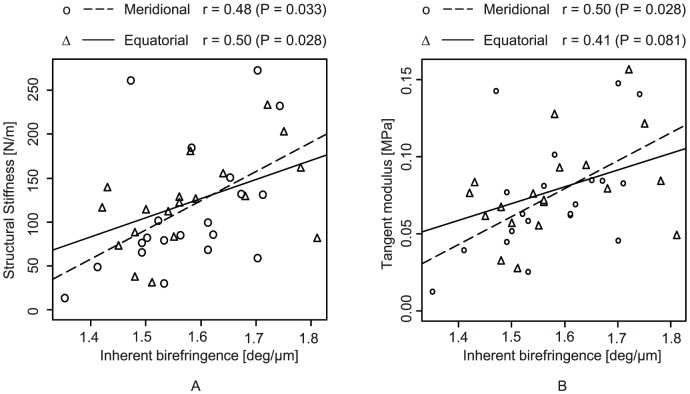
Plots of elastic parameters of structural stiffness (A) and tangent modulus (B) against inherent birefringence.

Figs. 6 (a) and (b) show data plots of the elastic parameters and inherent birefringence. A moderate positive correlation was found between the inherent birefringence and the structural stiffness (Pearson’s correlation coefficient, r  =  0.48, *P*  =  0.033 for meridional, and r  =  0.50 and *P*  =  0.028 for equatorial strain). A moderate positive correlation was also found between the inherent birefringence and the tangent modulus for meridional strain (r  =  0.52, *P* =  0.019). Although the statistical significance was low, the inherent birefringence and the tangent modulus for equatorial strain also showed moderate correlation (r  =  0.41 and *P*  =  0.081).

A moderate positive correlation was found between the birefringence at 6.5% strain and tangent modulus (r  =  0.58, *P*  =  0.008) and structural stiffness (r  =  0.53, *P*  =  0.017) for meridional strain. Although the statistical significance was low, the birefringence at 6.5% strain and each elastic parameters for equatorial strain also showed moderate correlation (tangent modulus; r  =  0.34, *P*  =  0.15, structural stiffness; r  =  0.42, *P*  =  0.075).

**Figure 7:**
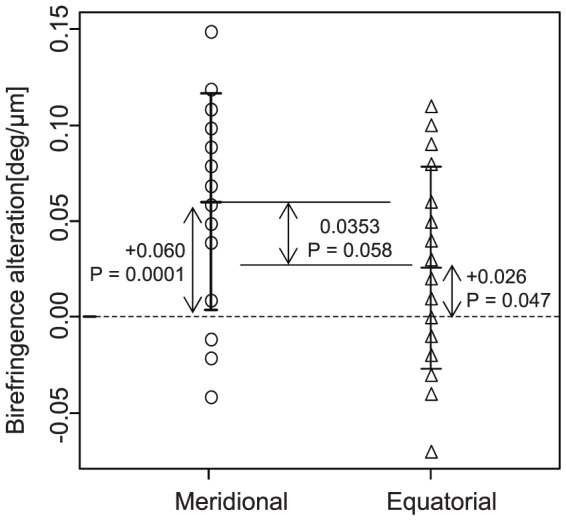
The birefringence alteration by meridional and equatorial strain.

The birefringence increased both by meridional (*P*  =  0.0001) and equatorial (*P*  =  0.047) strains as shown in Fig. 7. Marginal significance was found in the birefringence alteration between meridional and equatorial strains (*P*  =  0.058), where the mean birefringence elevation by meridional strain was higher than by equatorial strain. The birefringence alterations were +0.060 ± 0.056 deg/µm (mean ± standard deviation) for meridional and +0.026± 0.053 deg/µm for equatorial.

**Figure 8:**
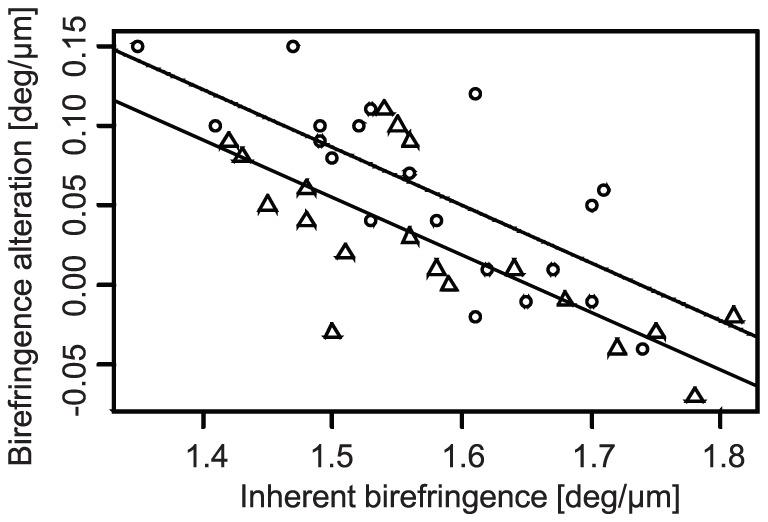
Birefringence alteration plotted against internet birefringence. Circles and rectangles indicate meridional and equatorial strain, respectively. The dashed and solid lines respectively represent regression curves of meridional and equatorial strains obtained by linear regression analysis which explains the birefringence alteration as a linear function of inherent birefringence and strain direction.

In order to analyze properties of birefringence alteration by strain, analysis of covariance (ANCOVA) was used in which an explained variable was the birefringence alteration and explanatory variables were the strain direction and the inherent birefringence as shown in Fig. 8. It was found that the most significant explanatory variable was the inherent birefringence (*P* < 0.0001) and the secondary explanatory variable was the strain direction (*P*  =  0.013). Since no significant interaction between the inherent birefringence and strain direction was found by preceded analysis of variance (ANOVA) (*P*  =  0.42), a linear regression was applied with assumption of an absence of interaction as shown in Fig. 8.

Additional correlation analysis revealed a negative correlation between the inherent birefringence and the alteration of the birefringence (Pearson’s correlation coefficient, r  =  -0.77, *P* < 0.0001 for meridional, and r  =  -0.72, *P*  =  0.0005 for equatorial).

## Discussion

Tangent modulus and structural stiffness of the sclera were positively correlated with the inherent birefringence, a result consistent with our previous study [15]. Collagen is the most important factor at sclera in relating birefringence to mechanical properties, because the highly organized collagen would result in high mechanical stiffness and resulting high birefringence.

**Figure 9:**
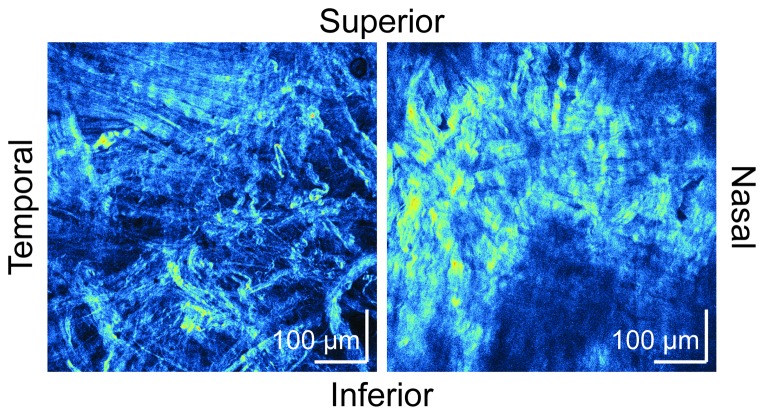
Second-harmonic-generation microscopy images of porcine sclera. The images were taken by a custom-made second-harmonic-generation microscope with an excitation wavelength of 800 nm and excitation pulse duration of 100 fs. The scale bars indicate 100 µm times 100 µm.

The unpaired t-test and ANCOVA indicated the strain direction dependency of the alteration of the birefringence, i.e., the elevation of birefringence was higher with the meridional strain. A previous study of human skin elasticity, which has more well organized collagen fibers than sclera, showed that skin dermal birefringence is altered by stretching or shrinking [25]. The study showed that stretching along the direction of relaxed skin tension lines (RSTL), to which the dermal collagen bundles are aligned, enhanced birefringence. However, stretching in a perpendicular direction to RSTL, and hence perpendicular to the collagen fiber bundle alignment, decreased the dermal birefringence. The authors suggested that the strain along the dermal collagen enhanced the alignment of the collagen fibers, and hence enhanced the dermal birefringence. In a similar manner, a strain perpendicular to collagen fibers destroyed alignment of collagen fibers, and reduced dermal birefringence [25]. In the case of sclera, it is known that collagen fibers are highly organized and aligned along a circumferential ring around the scleral canal in the peripapillary region [10], [12]. Collagen fibers in the peripheral sclera are more irregularly arranged to form interwoven lamellae, and they exhibit a wide range of diameters, which vary depending on thickness [11], [13], [14]. Most importantly, in contrast to the skin dermis, the region we measured (5-mm apart from the ONH) might have relatively randomly oriented collagen fibers. The second-harmonic-generation (SHG) microscopic images of porcine sclera (Fig. 9), which were obtained by a custom-made SHG microscope with an excitation wavelength of 800 nm and pulse duration of 100 fs [26], exemplified the variation of collagen fibers. A similar characteristic has also been known with rat sclera [27]. With this random collagen structure, the strain in both directions would promote alignment of collagen fibers and increase the birefringence. This would explain our results of birefringence elevation by the strains in both directions.

It is said that collagen fibers become uncrimped by stretch[28]. This uncrimping of collagen provides flexibility to the sclera and is believed to provide protection against IOP elevation[29]. At the same time this uncrimping of the collagen enhances the alignment of collagen fibers, and increased the birefringence.

The strain-directional dependency of the elevation of the birefringence would suggest that collagen was meridionally aligned at the region we examined, although further detailed study is necessary to confirm this hypothesis.

The negative correlation between inherent birefringence and birefringence alteration indicates that higher inherent birefringence tends to have smaller birefringence alteration by strain. This negative correlation is explained as follows. The high inherent birefringence is indicative of highly aligned collagen fibers before applying strain. If collagen has already been well aligned before applying strain, an additional increase of alignment by the strain is limited, and hence the birefringence alteration is only moderate.

It should be noted that, in the birefringence analysis, we have assumed the birefringence was uniformed throughout the sample. However, in practice, the birefringence would have spatial variations. This failure of assumption would results in degradation of reliability of the birefringence quantification. Further development of a data analysis method which accounts for this variation of tissue birefringence will provide us more accurate scleral birefringence analysis.

Our eventual goal is to use the birefringence measurement to assess the biomechanics of human sclera *in vivo*. In this study, the birefringence was found to be altered by applying stain. Furthermore, the birefringence alteration depended on the strain direction and inherent birefringence. This implies that birefringence of a sclera *in vivo* would be expected to be affected not only by its mechanical property, but also by intraocular pressure and the shape of the eye globe. This study and future systematic studies will hopefully further characterize the relationship between scleral biomechanics and birefringence, with the eventual goal of making PS-OCT a valuable tool for the clinical diagnosis of glaucoma.

## References

[1] Foster A, Resnikoff S (2005) The impact of Vision 2020 on global blindness. Eye 19: 1133–1135 doi:10.1038/sj.eye.6701973. 16304595 10.1038/sj.eye.6701973

[2] Sevim MS, Buttanri B, Acar BT, Kahya A, Vural ET, et al.. (2012) Ability of Fourier-domain Optical Coherence Tomography to Detect Retinal Ganglion Cell Complex Atrophy in Glaucoma Patients. J Glaucoma. Available: http://www.ncbi.nlm.nih.gov/pubmed/22407395. Accessed 2012 Jun 14. 10.1097/IJG.0b013e31824d1f97 22407395

[3] Quigley HA, Dunkelberger GR, Green WR (1989) Retinal ganglion cell atrophy correlated with automated perimetry in human eyes with glaucoma. Am J Ophthalmol 107: 453–464. 2712129 10.1016/0002-9394(89)90488-1

[4] Quigley HA, Katz J, Derick RJ, Gilbert D, Sommer A (1992) An evaluation of optic disc and nerve fiber layer examinations in monitoring progression of early glaucoma damage. Ophthalmology 99: 19–28. 1741133 10.1016/s0161-6420(92)32018-4

[5] Sommer A, Katz J, Quigley HA, Miller NR, Robin AL, et al (1991) Clinically Detectable Nerve Fiber Atrophy Precedes the Onset of Glaucomatous Field Loss. Arch Ophthalmol 109: 77–83 doi:10.1001/archopht.1991.01080010079037. 1987954 10.1001/archopht.1991.01080010079037

[6] Huang D, Swanson EA, Lin CP, Schuman JS, Stinson WG, et al (1991) Optical Coherence Tomography. Science 254: 1178–1181 doi:10.1126/science.1957169. 1957169 10.1126/science.1957169 PMC4638169

[7] Burgoyne CF, Crawford Downs J, Bellezza AJ, Francis Suh J-K, Hart RT (2005) The optic nerve head as a biomechanical structure: a new paradigm for understanding the role of IOP-related stress and strain in the pathophysiology of glaucomatous optic nerve head damage. Prog Retin Eye Res 24: 39–73 doi:10.1016/j.preteyeres.2004.06.001. 15555526 10.1016/j.preteyeres.2004.06.001

[8] Keeley FW, Morin JD, Vesely S (1984) Characterization of collagen from normal human sclera. Exp Eye Res 39: 533–542 doi:10.1016/0014-4835(84)90053-8. 6519194 10.1016/0014-4835(84)90053-8

[9] Christiansen DL, Huang EK, Silver FH (2000) Assembly of type I collagen: fusion of fibril subunits and the influence of fibril diameter on mechanical properties. Matrix Biol 19: 409–420 doi:10.1016/S0945-053X(00)00089-5. 10980417 10.1016/s0945-053x(00)00089-5

[10] Hernandez MR, Luo XX, Igoe F, Neufeld AH (1987) Extracellular matrix of the human lamina cribrosa. Am J Ophthalmol 104: 567–576. 3318474 10.1016/0002-9394(87)90165-6

[11] Komai Y, Ushiki T (1991) The Three-Dimensional Organization of Collagen Fibrils in the Human Cornea and Sclera. Invest Ophthalmol Vis Sci 32: 2244–2258. 2071337

[12] Yan D, McPheeters S, Johnson G, Utzinger U, Vande Geest JP (2011) Microstructural Differences in the Human Posterior Sclera as a Function of Age and Race. Invest Ophthalmol Vis Sci 52: 821–829 doi:10.1167/iovs.09-4651. 21051726 10.1167/iovs.09-4651 PMC3262314

[13] Summers Rada JA, Shelton S, Norton TT (2006) The sclera and myopia. Exp Eye Res 82: 185–200 doi:10.1016/j.exer.2005.08.009. 16202407 10.1016/j.exer.2005.08.009

[14] Watson PG, Young RD (2004) Scleral structure, organisation and disease. A review. Exp Eye Res 78: 609–623 doi:10.1016/S0014-4835(03)00212-4. 15106941 10.1016/s0014-4835(03)00212-4

[15] Yamanari M, Ishii K, Fukuda S, Lim Y, Duan L, et al.. (2013) Optical rheology of porcine sclera by birefringence imaging. PLoS ONE. In press. 10.1371/journal.pone.0044026 PMC3435379 22970158

[16] Lim Y, Yamanari M, Fukuda S, Kaji Y, Kiuchi T, et al (2011) Birefringence measurement of cornea and anterior segment by office-based polarization-sensitive optical coherence tomography. Biomed Opt Express 2: 2392–2402 doi:10.1364/BOE.2.002392. 21833376 10.1364/BOE.2.002892 PMC3149537

[17] De Boer JF, Milner TE (2002) Review of polarization sensitive optical coherence tomography and Stokes vector determination. J Biomed Opt 7: 359–371 doi:doi:10.1117/1.1483879. 12175285 10.1117/1.1483879

[18] Eilaghi A, Flanagan JG, Tertinegg I, Simmons CA, Wayne Brodland G, et al (2010) Biaxial mechanical testing of human sclera. Journal of Biomechanics 43: 1696–1701 doi:10.1016/j.jbiomech.2010.02.031. 20399430 10.1016/j.jbiomech.2010.02.031

[19] Elsheikh A, Geraghty B, Alhasso D, Knappett J, Campanelli M, et al (2010) Regional variation in the biomechanical properties of the human sclera. Exp Eye Res 90: 624–633 doi:10.1016/j.exer.2010.02.010. 20219460 10.1016/j.exer.2010.02.010

[20] Downs JC, Roberts MD, Burgoyne CF (2008) The Mechanical Environment of the Optic Nerve Head in Glaucoma. Optom Vis Sci 85: 425–435 doi:10.1097/OPX.0b013e31817841cb. 18521012 10.1097/OPX.0b013e31817841cb PMC2714589

[21] Makita S, Yamanari M, Yasuno Y (2010) Generalized Jones matrix optical coherence tomography: performance and local birefringence imaging. Opt Express 18: 854–876 doi:10.1364/OE.18.000854. 20173907 10.1364/OE.18.000854

[22] Lu S-Y, Chipman RA (1994) Homogeneous and inhomogeneous Jones matrices. J Opt Soc Am A 11: 766–773 doi:10.1364/JOSAA.11.000766.

[23] Duan L, Makita S, Yamanari M, Lim Y, Yasuno Y (2011) Monte-Carlo-based phase retardation estimator for polarization sensitive optical coherence tomography. Opt Express 19: 16330–16345 doi:10.1364/OE.19.016330. 21934997 10.1364/OE.19.016330

[24] G tzinger E, Pircher M, Geitzenauer W, Ahlers C, Baumann B, et al (2008) Retinal pigment epithelium segmentation bypolarization sensitive optical coherencetomography. Opt Express 16: 16410–16422 doi:10.1364/OE.16.016410. 18852747 10.1364/oe.16.016410 PMC2976032

[25] Sakai S, Yamanari M, Lim Y, Nakagawa N, Yasuno Y (2011) In vivo evaluation of human skin anisotropy by polarization-sensitive optical coherence tomography. Biomed Opt Express 2: 2623–2631 doi:10.1364/BOE.2.002623. 21991553 10.1364/BOE.2.002623 PMC3184871

[26] Yasui T, Takahashi Y, Ito M, Fukushima S, Araki T (2009) Ex vivo and in vivo second-harmonic-generation imaging of dermal collagen fiber in skin: comparison of imaging characteristics between mode-locked Cr:forsterite and Ti:sapphire lasers. Appl Opt 48: D88–D95 doi:10.1364/AO.48.000D88. 19340128 10.1364/ao.48.000d88

[27] Girard MJA, Dahlmann-Noor A, Rayapureddi S, Bechara JA, Bertin BME, et al (2011) Quantitative Mapping of Scleral Fiber Orientation in Normal Rat Eyes. IOVS 52: 9684–9693 doi:10.1167/iovs.11-7894. 10.1167/iovs.11-7894 22076988

[28] Fung YC, Cowin SC (1994) Biomechanics Mechanical Properties of Living Tissues,. 2nd ed. Journal of Applied Mechanics 61: 1007 doi:10.1115/1.2901550.

[29] Girard MJA, Suh J-KF, Bottlang M, Burgoyne CF, Downs JC (2009) Scleral Biomechanics in the Aging Monkey Eye. IOVS 50: 5226–5237 doi:10.1167/iovs.08-3363. 10.1167/iovs.08-3363 PMC2883469 19494203

